# The Effects of Hemoadsorption on the Kinetics of Antibacterial and Antifungal Agents

**DOI:** 10.3390/antibiotics11020180

**Published:** 2022-01-29

**Authors:** Giorgio Berlot, Stefano Di Bella, Ariella Tomasini, Erik Roman-Pognuz

**Affiliations:** 1 Azienda Sanitaria Universitaria Giuliano-Isontina, Department of Anesthesia and Intensive Care Medicine, Cattinara University Hospital, 34100 Trieste, Italy; ariella.tomasini@asugi.sanita.fvg.it (A.T.); erik.romanpognuz@asugi.sanita.fvg.it (E.R.-P.); 2 Clinical Department of Medical, Surgical and Health Sciences, Infectious Diseases Unit, Trieste University Hospital, 34100 Trieste, Italy; stefano932@gmail.com

**Keywords:** septic shock, mediators, hemoadsorption, antibiotics

## Abstract

The extracorporeal elimination of a pathogen or damage-associated molecular pattern via blood purification techniques is increasingly being used in patients with septic shock and other clinical conditions characterized by a life-threatening inflammatory response. The removal of these substances can be accomoplished by means of ultrafiltration or hemoadsorption. Independently from the blood putification technique used, they could also affect the clearance of antibacterial and antifungal agents with a potentially significant clinical impact. In our review, we describe the basic principles of ultrafiltration and hemoadsorption, the available devices for this latter and the existing experimental and clinical studies; the final paragraph is dedicated to practical considerations that can help clinicians to consider the clearance of antibiotics and antifungals attributable to these techniques to minimize the risk of a iatrogenic underdosage.

## 1. Introduction

Even in the absence of randomized clinical trials (RCTs) fulfilling the Evidence-Based Medicine (EBM) criteria or recommendations provided by the recently issued guidelines of the Surviving Sepsis Campaign (SSC) [1,2], the extracorporeal removal of pathogen or damage-associated molecular patterns (PAMPs and DAMPs, respectively) via techniques of blood purification (BP) is increasingly used to treat patients with septic shock or other clinical conditions characterized by an excessive inflammatory response [3]. This non-EBM founded popularity can be ascribed primarily to the failure of other approaches aiming at the neutralization of these substances by means of specific antibodies or inhibitors; actually, the results of many RCTs performed to evaluate the effect of this approach did not confirm the promising results obtained in experimental and pre-clinical studies. Different factors could account for these findings, including (a) the heterogeneity of the underlying conditions and of the related frailties of patients commonly encountered in the real-world scenario; and (b) the array of septic mediators that constitute a network rather than a cascade, making the blockade of a single cytokine unable to neutralize those located downstream. Then, it was hypothesized that their extracorporeal removal using BP could constitute a valid alternative. Basically, this goal can be accomplished via (a) their removal from the bloodstream by ultrafiltration (UF) through a filter that has pores with a cut-off value to allow the passage of substances with a molecular weight (MW) of septic mediators (50–70 kD), or (b) their adhesion to the surface of a material able to adsorb them: hemoadsorption (HA) [3,4]. 

Even if the UF and HA-based techniques share some similarities, such as the need for anticoagulation and a time-dependent decay of the clearance capabilities, the factors involved in the intensity of treatment are different and include (a) the volume of ultrafiltrate produced (Qf) per unit of time for the UF and (b) the volume of blood processed per unit of time the proxy of which is considered the blood flow (Qb).

Moreover, as both approaches are based on the interaction between the Qb and the filtering or sorbent material, it appears that a larger surface of contact or a longer duration of the procedure can be clinically relevant [3]. As far as the UF is considered, it has been hypothesized that higher Qf values could be associated with an improved survival of septic shock patients treated with UF; however, despite the encouraging results of some studies [5], a large RCT comparing elevated (70 mL/kg/h) with normal (35 mL/kg/h) Qf failed to confirm these findings [6] and this approach has been substantially abandoned; however, patients treated with higher Qf demonstrated a significant loss of antibiotics [7].

Whatever BP techniques are used, the possible influence on the pharmacokinetics (PK) of antibacterial, antifungal and antiviral agents constitutes a major concern. In fact, their removal is a recognized side-effect of UF, and a number of recommendations have been issued to adjust both the loading and maintenance doses [8], but less is known about the possible effect of the HA-based techniques. Should this effect exist also for them, it could at least partially account for the conflicting results observed in clinical studies involving septic shock patients treated by this approach: actually, although the use of HA has been associated with a decrease of the catecholamine requests and a better outcome in some studies [9,10,11,12,13], these findings have not been confirmed by other investigators [14,15,16,17], and a recent meta-analysis involving 120 patients treated with HA failed to establish clear positive or detrimental effects [18].

To address the issue of the possible removal of antibacterial and antifungal agents by means of HA we reviewed the available literature published in the last 10 years on PubMed using the key words “hemoadsorption” and “antibiotic removal”.

## 2. Principles of Hemoadsorption and Available Devices

HA can be performed either in a stand-alone mode or with Continuous Renal Replacement Therapy (CRRT) for patients with acute kidney injury (AKI) or with Extra Corporeal Membrane Oxygenation (ECMO); in the former case, it appears that the combination of the two techniques can exert additional effects on the removal of therapeutic agents. Three main HA techniques have been developed so far [7,19]. The first approach is based on a cartridge containing multiple polystyrene fibers covered by immobilized polymixin (Toraymixin, Toray Industries, Tokyo, Japan) that binds the endotoxin molecules. Due to this characteristic, its use has been advocated in the treatment of septic shock caused only by Gram-negative germs.

The second consists of a filter containing a modified AN69 membrane (oXiris, Baxter, Meyzieu, France) able to adsorb endotoxin and remove by means of UF several septic mediators while providing CRRT.

The last uses a cartridge containing polystyrene and divinylbenzene microbeads (Cytosorb^®^, Cytosorbents Corporation, New Jersey, USA; Aferetica s.r.l., Bologna, Italy) with a large surface (~40,000 m^2^), where both hydrophobic pro- and anti-inflammatory mediators with a MW of 5–60 kD are absorbed. The efficacy of Cytosorb^®^ is concentration-dependent, as substances present in large concentrations are removed more efficiently than those with lower levels. If needed, the Cytosorb^®^ can also be used with CRRT/ECMO.

## 3. Experimental Studies

Some nonclinical studies have addressed the pharmacokinetic (PK) of antibiotics during HA. With some minor differences, the general design of these investigations consists in the measurement of the concentration of antibiotics after a determined interval in the fluid bathing a material used for HA or in the fluid perfusing a cartridge either at its inlet and outlet.

Harm et al. studied a number of sorbent materials incubated with different classes of antibiotics at concentrations close to that recommended in septic shock patients. After 60 min, although all agents presented some variations, it was demonstrated that these materials were more marked in the batch containing activated charcoal in a device suited for use as a bridge for liver transplantation [20].

In an in vitro study using the Cytosorb^®^, König et al. demonstrated that (i) there was a wide pre-and post-cartridge concentration gradient of different antibiotic classes, including β-lactams, quinolones, aminoglycosides, glycopeptides and azoles dissolved in normal saline (NS) or human albumin 5% at therapeutic concentrations and pumped through the cartridge, indicating their adsorption; and that (ii) this effect was evident since the beginning of the procedure; and (iii) all the studied agents dropped to sub-therapeutic levels after 5–20 min of HA [21].

Schneider et al. studied two groups of pigs treated with the same resin and with a sham procedure during a 6 h period. They measured the blood concentration of 17 administered drug combinations similar to those clinically used; total clearance (Tc) and that part attributable only to the HA were then calculated. The impact of HA on Tc was the highest for fluconazole, linezolid and liposomal amphotericin B (+282, 115 and 75%, respectively) and lower for other agents (Table 1) [22]. Overall, lipophilic molecules appeared to be more avidly bound by the resin. However, as stated by the authors, this model hardly represented the real-world scenario of septic shock patients treated with HA: in these subjects other factors influenced the Tc, including the possible concomitant occurrence of an AKI requiring a CRRT, altered distribution volume associated with increased microvascular permeability, and possible competition with PAMPS; moreover, the duration of the observation was much lower than the running time of the cartridge according to the manufacturer’s indications.

More recently, Biever et al. measured the concentration of remdesivir and of its main active metabolite GS-441524 in a serum-perfusing Cytosorb^®^ cartridge and found that both agents were almost completely removed from the serum after only 60 min from the initiation of the procedure [23].

Taken together, all these studies demonstrated that many different antibiotic, antifungal and antiviral agents can be absorbed during a HA procedure with the Cytosorb^®^.

## 4. Clinical Studies

The clinical studies on the PK of antimicrobial agents in patients treated with HA basically consist of case reports or small series involving only a few patients. The study designs are represented by the assessment of the blood or plasma levels of the agents sampled at different intervals of time or upstream and downstream of the HA cartridge.

Plasma levels of clindamycin were measured in a septic shock patient treated with ECMO and Cytosorb^®^ by Poli et al., and the results were plotted against a theoretical curve of PD: the authors concluded that, even in the presence of a strong day-to-day variation of the drug, it was not significantly cleared by HA [24].

Dimski et al. measured the levels of vancomycin and teicoplanin three patients with septic shock treated with Cytosorb^®^ given in different timeframes: the two drugs were removed almost completely when they were administered in a 60 min infusion, and blood levels dropped to sub-therapeutic levels even when the adsorptive capabilities appeared to decay with time; conversely, the levels of vancomycin, even when decreased, remained in the therapeutic range when this agent was administered in a continuous infusion [25].

Zurl et al. demonstrated the plasma levels of the antifungal agent Isavuconazole considerably varied among patients not treated or treated with CRRT associated with ECMO or Cytosorb^®^: in this latter group, the measured levels of Isavuconazole were >70% less than in the former [26].

Khöler et al. measured the blood levels of linezolid given as a 60 min infusion at different time points (before, 15, 60, 120 and 480 min) in a postoperative patient treated with Cytosorb^®^ and demonstrated that the blood levels after 8 h were below the therapeutic range [27].

Very recently, Liebchen et al. demonstrated that, in a group of septic shock patients treated with meropemen, the Cytosorb^®^ associated with CRRT did not significantly remove the drug [28].

## 5. Practical Considerations

As stated above, many studies have been published on the effects of the Cytosorb^®^ and other HA-based techniques on septic shock patients. However, it is hard to draw definite conclusions since factors other than the BP can influence the outcome, including the timing of initiation, the intensity of the treatment, and the appropriateness of the antibiotic regimen. Actually, the use of HA could represent a double-edged sword. It appears that (i) different classes of anti-infective agents can be efficiently removed from the bloodstream; (ii) in many cases this effect is time-dependent, being maximal in the initial hours of the procedure when the binding sites are still largely unsaturated; and, more importantly, (iii) the combined effect of these two mechanism can determine a sub-optimal concentration of these substances just when their appropriate levels are keenly warranted. The SSC recommends appropriately prompt administration alongside a type of administration capable of maximizing the anti-infective effects (e.g., extended infusion for β-lactams) of antibiotics/antifungals [2] as the cornerstone for treating sepsis and septic shock. This effect and the related consequences could be even more relevant should the cartridge be changed before its exhaustion to take advantage of its binding capabilities to septic mediators as suggested by some authors [29].

Although precise indications are lacking, different approaches can be used to limit the risk and obtain a personalized antibiotic treatment. First, although the bulk of the available data derives from experimental studies under conditions far from clinical, it is mandatory to know if, and in what amount, the used agent is removed (Table 2). Second, the use of HA in combination with CRRT can further increase the elimination of the anti-infective agents compared to HA alone. Third, the drug removal is maximal in the initial phase of HA and decreases with time due to the saturation of the binding sites; thus, it could be advisable to administer a loading dose followed by a continuous infusion later on. Third, therapeutic drug monitoring (TDM) before the initiation of the following HA session and to adjust the dose accordingly is warranted. Last, but not the least, although the efficacy of HA has not been definitely demonstrated, and the results of clinical investigations are somewhat contrasting, in most studies the relevant factors such as the intensity of treatment and the time of initiation are reported. As demonstrated by Berlot et al. [30] it is possible that optimizing the amount of blood processed and reducing the treatment-free interval could represent a key factor for a better outcome similar to other interventions recommended in the SSC [30].

## 6. Conclusions

Different BP techniques are currently used to treat clinical conditions characterized by an overwhelming inflammatory reaction. It should be recognized that anti-infective agents can be removed along with the mediators; thus, the use of TDM and the adjustment of the dosing regimens of anti-infective drugs is warranted.

## Figures and Tables

**Table 1:** Additional clearance of anti-infective agents provided by Cytosorb^®^; from Schneider AG et al., modified.

Agent	Variation (%)
Liposomal Amphotericin B	74.9
Anidulafungin	22.7
Cefepime	1.2
Ceftriaxone	5.2
Ciprofloxacin	14.5
Clarithromycin	4.7
Clindamycin	6.4
Flucloxacillin	15.9
Fluconazole	282.2
Linezolid	114.6
Meropenem	6.3
Metronidazole	15.4
Piperacillin	19.4
Posaconazole	32.0
Teicoplanin	30.7
Tobramycin	5.5

**Table 2:** Effect of HA with Cytosorb^®^ on the PK of different anti-infective agents. The removal was considered significant when the concentration of the challenged agent dropped to sub-therapeutic levels.

Agent	Mode	Administration	Removal	Reference
Clindamycin	Associated with ECMO and CRRT	Intermittent	Not significant	[26]
Isavuconazole	Associated with CRRT	Intermittent	Significant	[28]
Linezolid	Associated with CRRT	Intermittent	Significant after 4 h	[29]
Meropenem	Stand alone and associated with CRRT	Intermittent	Not significant	[30]
Teicoplanin + Vancomycin	Stand Alone	Intermittent	Significant	[27]
Vancomycin	Stand Alone	Continuous Infusion	Not significant	[27]

CRRT: continuous renal replacement therapy; ECMO: extracorporeal membrane oxygenation.
